# Chromosome preference of disease genes and vectorization for the prediction of non-coding disease genes

**DOI:** 10.18632/oncotarget.20481

**Published:** 2017-08-24

**Authors:** Hui Peng, Chaowang Lan, Yuansheng Liu, Tao Liu, Michael Blumenstein, Jinyan Li

**Affiliations:** ^1^ Advanced Analytics Institute & Centre for Health Technologies, University of Technology Sydney, Broadway, NSW, Australia; ^2^ Centre for Childhood Cancer Research, University of New South Wales, Sydney, Kensington, NSW, Australia; ^3^ School of Software, University of Technology Sydney, Broadway, NSW, Australia

**Keywords:** chromosome preference, vectorization, long noncoding RNA

## Abstract

Disease-related protein-coding genes have been widely studied, but disease-related non-coding genes remain largely unknown. This work introduces a new vector to represent diseases, and applies the newly vectorized data for a positive-unlabeled learning algorithm to predict and rank disease-related long non-coding RNA (lncRNA) genes. This novel vector representation for diseases consists of two sub-vectors, one is composed of 45 elements, characterizing the information entropies of the disease genes distribution over 45 chromosome substructures. This idea is supported by our observation that some substructures (e.g., the chromosome 6 p-arm) are highly preferred by disease-related protein coding genes, while some (e.g., the 21 p-arm) are not favored at all. The second sub-vector is 30-dimensional, characterizing the distribution of disease gene enriched KEGG pathways in comparison with our manually created pathway groups. The second sub-vector complements with the first one to differentiate between various diseases. Our prediction method outperforms the state-of-the-art methods on benchmark datasets for prioritizing disease related lncRNA genes. The method also works well when only the sequence information of an lncRNA gene is known, or even when a given disease has no currently recognized long non-coding genes.

## INTRODUCTION

Benefiting from the breakthroughs of the next generation sequencing (NGS) technologies [1], disease related protein coding genes have been widely studied during the last decades. Non-coding genes have been also found involved in human disease development by functioning as regulators of their target protein coding genes [2]. Especially, long non-coding RNAs (lncRNAs), a kind of RNA that do not encode proteins and are longer than 200nt, have been found to contain significant genetic information and functions [3]. The dysregulation of lncRNAs can result in the dysfunction of their target protein coding genes or their participated cellular processes, causing the development of diseases. For example, the lncRNA NEAT1 was reported to be a potential target of ERα and is an important mediator for maintenance of prostate cancer [4]. Loc285194, another lncRNA, is also a tumor suppressor that regulates p53 [5]. Increasing number of studies have been focusing on the application of disease-lncRNA associations including disease diagnosis [6], survival prediction [7] and RNA therapeutics [8]. However, the function annotation of lncRNA genes such as their roles in disease development is remaining largely unknown.

Genomic locus inferring methods [9–10], computational methods including gene-lncRNA co-expression methods [11–12], network analysis methods [13], similarities analysis or semi-supervised learning methods [14], supervised learning methods [15] and others [16] can speed up this area of research for disease gene prediction. The network analysis heavily relies on the topology properties of the constructed networks. The semi-supervised learning methods depend on accurate similarity measurements between diseases and lncRNAs. The supervised learning approach has not been extensively explored because of lack of reliable negative samples of disease related lncRNA genes.

We propose to use a positive-unlabeled learning (PU-learning) method to predict disease related lncRNA genes. PU learning can well address the problem of lacking reliable negative samples to gain high prediction performance. In this work, we also introduce a novel vector $<V_{d}>$ to represent a disease *d*, and a novel vector $<V_{Lnc}>$ to represent an lncRNA gene *Lnc*. We merge these two vectors as $<V_{d},V_{Lnc}>$ to represent the pair of disease *d* and the lncRNA gene *Lnc*. The prediction problem is: whether this merged vector can be mapped to 1 or 0 with a certain level of probability. If it is mapped to 1 with a high probability (e.g., 90%), then it means that the disease *d* is related to the lncRNA gene *Lnc* under a high probability. Otherwise, the disease *d* has little relationship with lncRNA gene *Lnc*.

The novel disease vector representation $<V_{d}>$ consists of two sub-vectors. The elements of the first sub-vector $<V_{d}^{chr}>$ represent the chromosome substructures’ distribution information entropies of the genes related to the disease *d*. We consider 45 chromosome substructures in this work (details presented later).

This idea for disease representation is inspired by a chromosome substructure enrichment analysis of the disease related protein coding genes. It is similar to gene pathway enrichment analysis that the protein gene set of a disease can be enriched at each chromosome substructure containing the protein gene set. We have observed that about 16.2% of 2802 diseases' genes can be enriched to chromosome 6 p-arm (with Fisher's exact test, p-value<0.05), implying a strong chromosome preference of disease genes. This preference is significantly higher than the second most enriched chromosome 2 q-arm (containing just 5.92% of the 2802 diseases). Furthermore, no disease gene set can be enriched to the chromosome 21 p-arm. Our hypothesis is that genes are located at various positions on chromosomes and mitochondrion, and the distribution of disease related protein coding genes on the chromosomes can be used to characterize the differences between diseases.

The second sub-vector $<V_{d}^{path}>$ represents the KEGG pathway groups’ distribution information entropies of disease *d* related genes enriched KEGG pathways. Human KEGG pathways [17] can be divided into 30 groups. By the disease gene KEGG pathway enrichment analysis on the 2802 diseases, we have observed that almost all these KEGG pathways are involved in disease developments. The distribution of disease gene sets on KEGG pathway groups is also uneven. For example, more than 30% of the 2802 diseases are associated with 6 pathways including hsa04933: AGE-RAGE signaling pathway in diabetic complications and hsa05321: Inflammatory bowel disease (IBD). In comparison, as many as 61 kinds of pathways are related to less than 1% of these diseases.

Comparing with existing disease characterization methods through computing similarities of disease related coding or non-coding genes [18], semantics [19], phenotypes [20, 21], symptoms [22] and ontology [23], our disease vectorization $<V_{d}^{chr},V_{d}^{path}>$ is much simpler. It does not need repeated set operations such as union and intersection or large scale of text mining. Our disease vectors are also effective to capture unique disease characteristics. The disease similarity can reach to the average area under ROC curve (AUC) of 0.9458 when the diseases are represented by our vectors. However, FunSim [18] and a disease symptom representation method [22] have only 0.9202 and 0.7674 AUC respectively on the same set of diseases.

**Figure 1:**
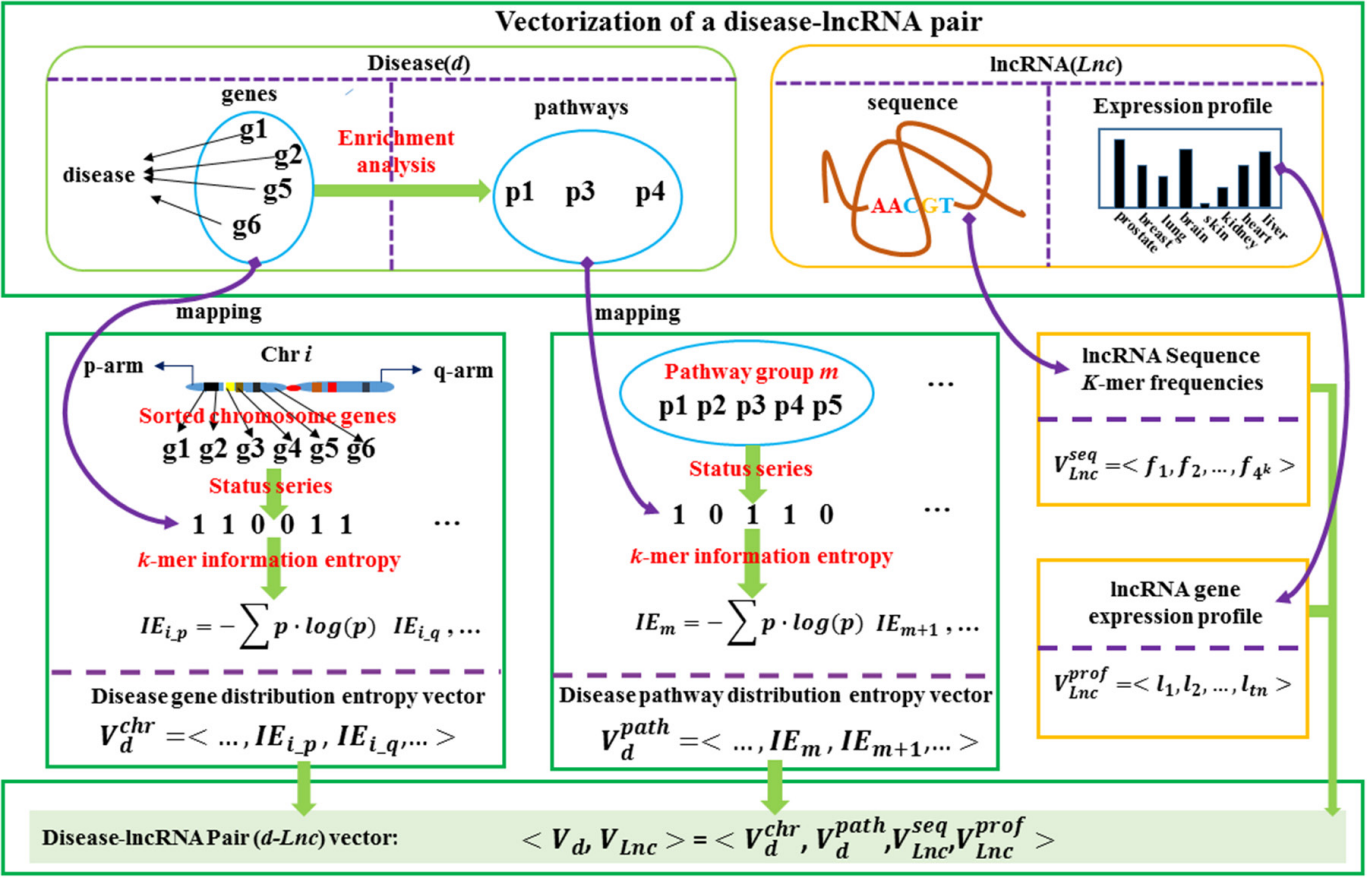
The flowchart for the vectorization representation of a disease-lncRNA gene pair A disease-lncRNA gene pair can be represented by the integration of four sub-vectors including disease gene chromosome substructures’ distribution information entropy vector (disease gene distribution vector), the disease gene enriched pathway groups’ distribution information entropy vector (disease pathway distribution vector), the lncRNA gene sequence’s *k*-mer frequency vector and the lncRNA gene expression profile.

The vector $<V_{Lnc}>$ representing an lncRNA gene *Lnc* consists of two sub-vectors $<V_{Lnc}^{seq}>$ and $<V_{Lnc}^{prof}>$ as well.The first one represents its sequence’s *k*-mer frequencies, and the second one represents its expression profiles. Merging the two disease sub-vectors $<V_{d}^{chr}>$ and $<V_{d}^{path}>$, the two lncRNA sub-vectors $<V_{Lnc}^{seq}>$and $<V_{Lnc}^{prof}>$, we can represent a disease-lncRNA gene pair (denoted *d-Lnc*) as $<V_{d},V_{Lnc}>$ Procedures for constructing the main sub-vectors are shown in Figure 1.

Disease related lncRNA genes should also prefer to co-expressing with other genes that are associated with this disease (such as those lncRNA genes which regulate some of the disease related protein coding genes). With this hypothesis, we add these co-expression features as the fifth sub-vector $<V_{co-\exp}>$ to the merged vector $<V_{d},V_{Lnc}>$. From our baseline classifier selection experiments, we have proved that this new sub-vector can further improve the prediction performance.

A bagging SVM for PU learning algorithm [24] is adopted to prioritize disease related lncRNA genes. This model was trained on a set of disease-lncRNA vectors. On three data sets retrieved from three disease-lncRNA association databases: LncRNADisease [9], Lnc2Cancer [25] and MNDR [26], the overall AUC scores of leave-one-out cross validation (LOOCV) by our method are 0.8016, 0.8335 and 0.7527 respectively. This performance is significantly superior to two state-of-the-art methods: LRLSLDA [14] (0.6882, 0.7308 and 0.6346) and LRLSLDA-ILNCSIM [27] (0.6949, 0.7390 and 0.6435). Especially when only the sequence information of the lncRNA genes is available, our method can still work well for the prediction. The overall LOOCV AUC scores for the three datasets are 0.7889, 0.8266 and 0.7216. The results of the following leave-one-disease-out cross-validation (LODOCV) experiments show the ability of our method to predict without known disease related lncRNA genes for a given disease as the average AUC value is 0.7356 for the LncRNADisease dataset. There are 68 out of 162 diseases can achieve the AUC values bigger than 0.9.

## RESULTS

### Chromosome preference and disfavor of disease genes

In the understanding of unique characteristics of disease genes on the chromosomes, we constructed chromosome enrichment analysis of disease genes. The process is similar to the implementation of Fisher’s exact test for pathway enrichment analysis which we have described in Algorithm 1. The main difference is that the pathway genes are replaced with the chromosome involved genes. We note that only protein coding genes are considered for the chromosome preference analysis of disease genes as the non-coding disease genes are under prediction.

**Algorithm 1:** Disease vectorization

**Input** disease *d* related gene set *d*_*g*_, Approved genes *G*, human pathway set *P*, each pathway *p*_*j*_ contained gene set *g*_*pj*_, parameter *k1*, *k2*, *T*; 1: Sort *G* according to the chromosome location of *g*_*i*_, sort *P* according to the ids of *p*_*j*_; 2: Separate *G* according to the natural chromosome structure such as chr1 p-arm, chr1 q-arm,…. There are totally *S* chromosome substructures, *i.e. chr*_1_, *chr*_2_, …, *chr*_*u*_, …, *chr*_*S*_; 3: Divide *P* into *T* groups, *i.e. p*_*g*1_, *p*_*g*2_, …, *p*_*gw*_, …, *p*_*gT*_; 4: Generate the initial status series of each *S_chr*_*u*_ = (0,0, …, 0) and *S*_*p*_*gw*_ = (0, 0, …, 0); 5: Map *d*_*g*_ to *chr*_*u*_ according to its location and change the corresponding status in *S_chr*_*u*_ as 1; 6: Set *k* = *k1* **7: for *u* =** 1 **to** *S* **do** 8: Scan *S*_*chr*_*u*_ with window size of *k* and step size 1; 9: Compute the frequency of *q*th *k*-mer sub-status series as *f*_*q*_; 10: Compute the information entropy of the *k*-mer sub-status series for *S_chr*_*u*_ as $IE_{chr_{u}}=-f_{q}\times \sum_{q=1}^{2^{k}}\log\left(f_{q}\right);$ **11: end for** **12: for** *j* = 1 **to** *M* ***do*** 13: Count genes in *g*_*pj*_ as *L* = *Length (g*_*pj*_*)*; 14: Count genes mapped into *p*_*j*_ as $B=Length\left(d_{g}\cap g_{pj}\right);$ 15: Do the fisher’s exact test: 16: [h, p, stats] = *fishertest* ([*L*- *B*, *B*;*A* - *L* - *n* + *B*; *n* - *B*]), where *p* is the p-value; 17: **if** *p* <= 0.05 **then** 18: Change the status of *p*_*j*_ in *S*_*p*_*gw*_ as 1; **19: end if** **20: end for** 21: Set *k* = *k2* 22: **for** *w* = 1 **to** *T* **do** 23: Scan *S_p*_*gw*_ with window size of *k* and step size 1; 24: Compute the frequency of *v*th *k*-mer sub-status series as *f*_*v*_ 25: Compute the information entropy of the *k*- mer sub-status series for *S_p*_*gw*_ as $IE_{P}g_{w}=-f_{v}\times \sum_{v=1}^{2^{k}}log\left(f_{v}\right);$ **26: end for** **Output** The disease gene distribution entropy vector: $<{\text{V}}_{d}^{chr}>=<IE_{chr_{1}},IE_{chr_{2}},\ldots ,IE_{chr_{S}}>$ **Output** The disease pathway distribution entropy vector: $<V_{d}^{path}>=<IE_{pg_{1}},IE_{pg_{2}},\ldots ,IE_{pg_{r}}>$

**Figure 2:**
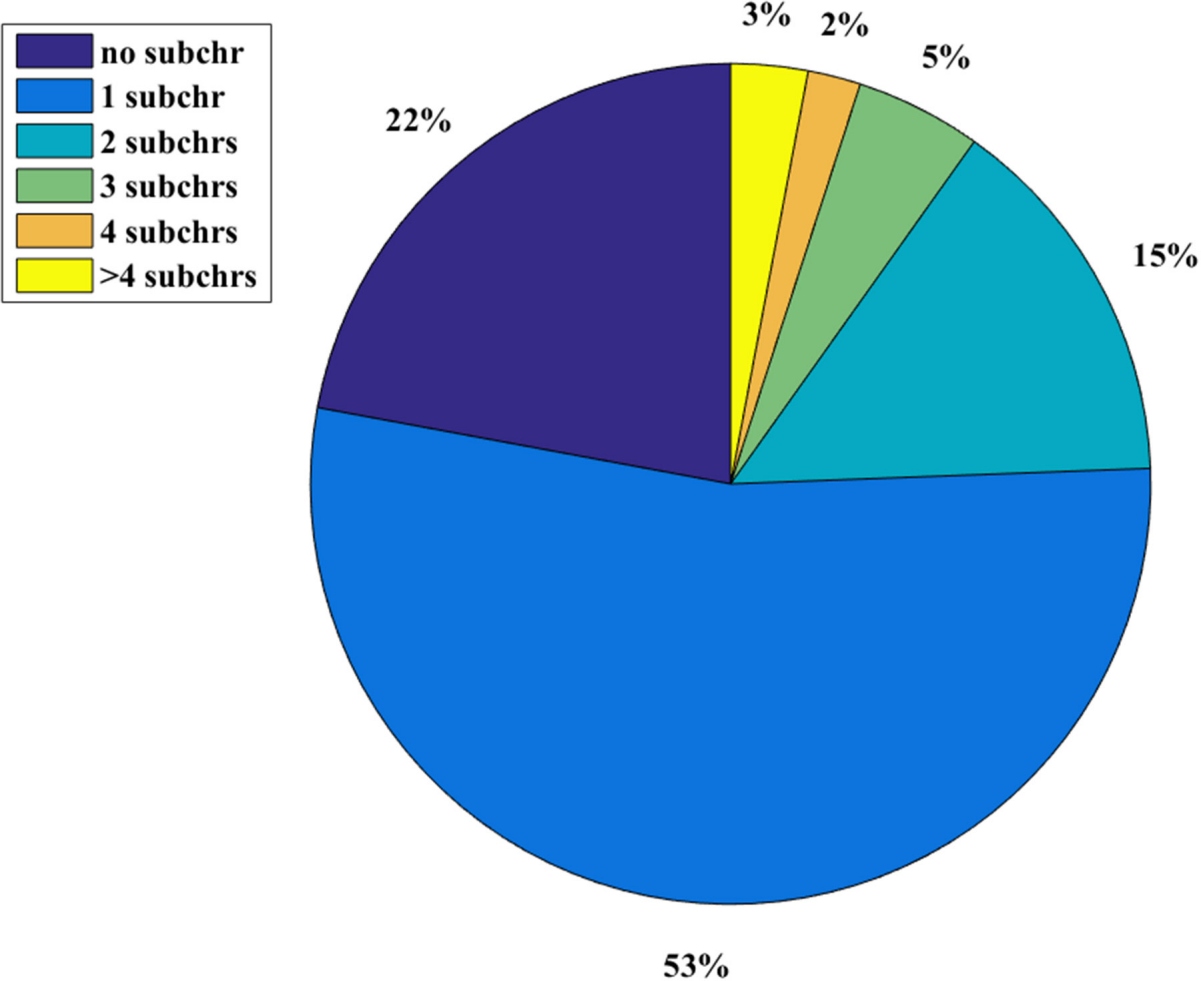
The disease chromosome enrichment analysis pie graph Subchr means chromosome substructure. We did the statistics of how many chromosomes a disease gene set enriches. More than a half (53%) of the 2802 diseases are just enriched to only one chromosome substructure, while just 3% of these diseases can be enriched to more than 4 chromosome substructures.

**Figure 3:**
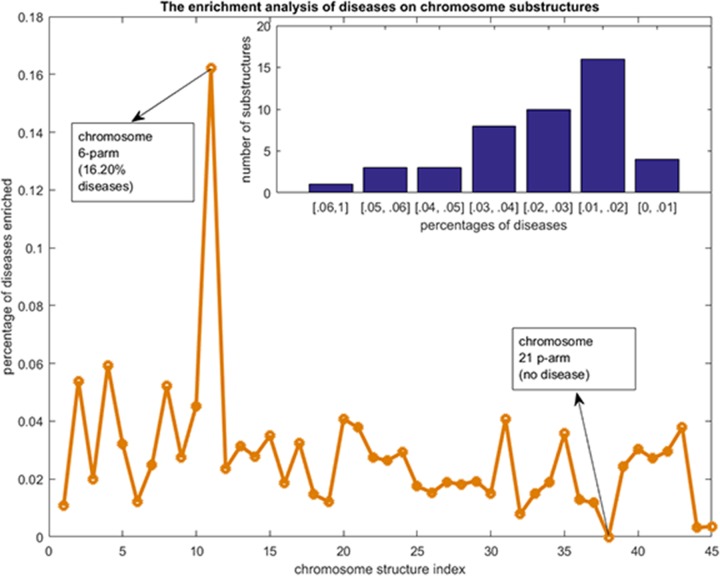
The disease chromosome enrichment analysis results The y-axis are percentages of diseases that enriched to each of the chromosome substructures. The x-axis are the indexes of the chromosome substructures. The bar graph at the top right shows the statistics of the numbers of chromosome substructures that contained by diseases with given percentages scopes.

The 24 chromosomes of human genome can be naturally divided into 48 substructures with the p-arm and the q-arm as two substructures for each chromosome. However, for chromosome 13 (chr13), there is only one protein gene on the centromere and there is no approved protein gene located at its p-arm; for chromosome 14, only one gene is located at its p-arm; and there is no gene located at the p-arm of chr15 or chr22. Thus, these four chromosomes were not divided. We consider the mitochondrion as a special chromosome which cannot be divided into two substructures. In total, we have 45 chromosome substructures, namely S=45 in Algorithm 1. Figure 2 and Figure 3 show the statistics of the chromosome substructure enrichment analysis for the disease genes of each of the 2802 diseases.

There are about 75% of the diseases whose related gene sets can be enriched to no more than 1 chromosome substructure (Figure 2). There are just 3% of the diseases whose related gene sets can be enriched to more than 4 chromosome substructures. This distribution of disease genes on the chromosome substructures reveals that the disease genes of a given disease are very likely located at a neighborhood region. As indicated by Figure 3, the p-arm of chromosome 6 is the most preferred substructure of disease genes - about 16.2% of the disease related gene sets can be enriched here. This percentage is significantly higher than the other substructures (all no more than 6%). Interestingly, there is no disease related gene set that can be enriched to the p-arm of chromosome 21. From the top-right bar graph of Figure 3, we can also see that 16 out of the 45 chromosome substructures are enriched by only 1% -2% of the 2802 gene sets. There are 10 and 8 substructures can be enriched by 2%-3% and 3%-4% of the 2802 gene sets. Thus, most of the chromosome substructures (38 out of 45) can be enriched by no more than 3% of the 2802 gene sets. These observations suggest a phenomenon that disease genes are unevenly distributed in the 45 chromosome substructures. The genes related to a disease are preferred at a physical neighborhood close to each other in the chromosomes. This observation of chromosome preference lays down the foundation for our disease vector representation.

We also conducted pathway enrichment analysis to understand the distribution of disease genes in human KEGG pathways. We found that disease genes are also unevenly enriched in these pathways. More than 30% of the 2802 diseases are associated with one of the top 6 pathways such as hsa04933: AGE-RAGE signaling pathway in diabetic complications, and hsa05321: Inflammatory bowel disease (IBD). In contrast, 61 out of 303 pathways are related to less than 1% of these diseases. More details are reported in Supplementary File 1.

### Performance on the prediction of highly similar diseases using our disease vector representation

We tested the performance of our vectorization model for computing disease similarities on the dataset downloaded from the supplementary files of Cheng's paper [18]. It contains a candidate disease set and a benchmark set of similar disease pairs. The disease set is composed of 2802 diseases and their related genes. There are 70 similar disease pairs in the benchmark set. Zhou *et al.* [22] proposed a symptom representation method for measuring disease similarities. To compare this method with ours, we downloaded their similarity scores between 1596 diseases and mapped these diseases to the 2802- disease set. Totally 1012 diseases and 56 similar disease pairs in the benchmark set can be mapped. These two disease sets have been stored in Supplementary File 2.

Following cheng's method, we drew a ROC curve to display how our method can rank the similar pairs in the benchmark set comparing with those randomly selected unknown disease pairs. That means, for a given threshold, if the similarity of a pair in the benchmark set exceeds this threshold, it is defined as a true positive, otherwise, as a false negative. Inversely, an unknown disease pair exceeds the threshold is defined as a false positive. A total of 560 testing disease-disease pairs were randomly selected from the 1012 candidate diseases (but not overlapping with the benchmark set). This process was repeated 100 times.

There are three parameters, i.e. *k*1, *k*2 and *T*, for Algorithm 1 and one parameter *θ* for equation (3) need to be tuned. According to the HGNC database, there are 19025 approved protein coding genes. Because the minimum length of the chromosome substructure is 9 (only 9 protein coding genes on this substructure), thus the parameter *k*1 was changed from 1 to 9 with the step size of 1. There are 303 different human KEGG pathways. To simplify our model, we set *T*=30 with the first 29 groups containing 10 pathways while the last group has 13 pathways. Finally, the disease genes chromosome substructures’ distribution information entropy (disease gene distribution entropy) feature is represented as a 45-dimensional vector while the disease gene enriched pathway groups’ distribution information entropy (disease pathway distribution entropy) feature is a 30-dimensional vector. *k*2 was changed from 1 to 10 with the step size of 1. The integration parameter *θ* was in the range of [0, 1].

When *k*1=9, we can get the biggest average AUC=0.9429. Meanwhile, when *k*2=8, the AUC value with just pathway distribution entropy vectors can achieve 0.8872. Thus, we set *k*1=9 and *k*2=8 for the subsequent experiments.

**Figure 4:**
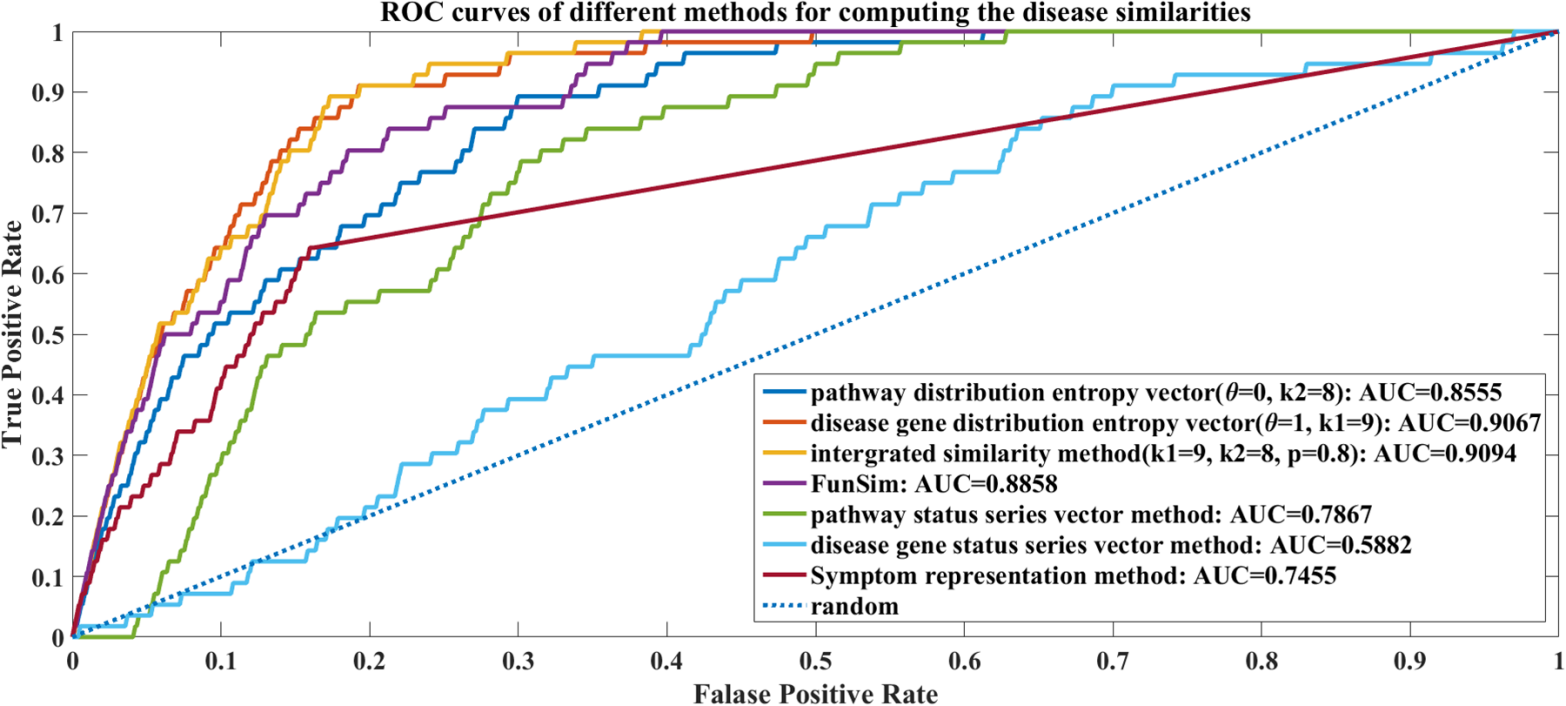
The ROC curves of different methods for computing the disease similarities There are 7 ROC curves: the disease pathway distribution entropy vector method (*θ*=0, AUC=0.8555); the disease gene distribution entropy vector method (*θ*=1, AUC=0.9067); the integrated similarity method (*θ*=0.8, AUC=0.9094); the pathway status series vector method (AUC=0.7867); the disease gene status series vector method (AUC=0.5882); FunSim (AUC=0.8858) and Symptom representation method (AUC=0.7455).

We also compared the performances of our methods (namely the entropy vector methods and the status series vector methods), the FunSim [18] and symptom representation method [22]. We implemented the FunSim according to the published paper. Then, the AUC values were computed according to the scores via different methods. During the comparison, *θ* was set to be 0 to 1 with the step size of 0.1. When *θ*=0.8, the integrated similarity method can work the best with average AUC=0.9458. We drew the corresponding overall ROC curves (all the 100 times repeat experiments’ results are combined together to compute the False Positive Rate and True Positive Rate; thus, the overall AUC values are smaller than the average AUC values) of the 100 times experiments in Figure 4. More comparison results for the original 2802 disease set can be found in the Supplementary File 1.

Figure 4 shows that the integrated similarity method is better than the other methods. However, it just improves 0.0027 on the AUC value comparing with just disease gene distribution entropy vector method (*k*1=9, *θ*=1). It implies that there are not much complementary between the disease gene distribution entropy and disease pathway distribution entropy features as to compute the similarities of diseases. The integrated similarity method with *θ*=0.8 outperforms the FunSim and symptom representation method by improving AUC values of 0.0236 and 0.1639 respectively. In comparison, the status series vector methods cannot work as well as the entropy vector methods. The entropy vector methods (disease gene distribution entropy *vs.* disease gene status series and disease pathway distribution entropy vs. pathway status series) improve the overall AUC values by 0.3185 and 0.0688. This proves our “part overcomes the whole” hypothesis that our dividing and information entropy strategy for representing diseases is more effective than the original status series.

### Performance on the prediction and prioritization of disease related lncRNA genes

The performance of our disease vectorization method for predicting and prioritizing disease related lncRNA genes was tested and evaluated on three data sets: the lncRNADisease dataset (454 positive samples, i.e., 454 known associations between some diseases and some lncRNA genes), the lnc2cancer dataset (594 positive samples) and the MNDR dataset (176 positive samples). See details of these data sets at the section Materials and Methods.

### Classifier and parameter selection for final prediction model with the lncRNADisease dataset

We used both liner and RBF kernel for the SVM-based positive-unlabled learning method to conduct cross validation on the lncRNADisease data set. The number of positive samples is 454, and the number of unlabeled samples (i.e., the number of unknown associations) is 29840, derived by exhaustively pairing the 162 diseases and 187 lncRNAs in the lncRNADisease data set after the deduction of the number of 454 positive samples. Recall that our vector representation for a pair of disease and lncRNA gene consists of five sub-vectors. Here, we choose different combination of these sub-vectors to understand that all of these sub-vectors are important for the prediction. The steps are presented in Algorithm 2.

**Algorithm 2:** A bagging SVM for prioritizing the disease related lncRNA genes

**Input** Positive dataset *PO*, unlabeled dataset *UN*, bootstrap sample size *R*, bootstrap number *V*, SVM parameters, feature type *W*; 1: **for *a*=**1 **to** 100 **do** 2: Randomly select |*PO*| of unlabeled samples as negative samples; 3: Implement a 5-fold cross validation on the positive-negative dataset with feature type *W* and do grid search of SVM parameters; 4: **end for** 5: Use F1 score as the metric, determine the optimal SVM parameters *opPara* and the optimal feature type *W*_*op*_; 6:∀ xϵUN,n(x)←0,f(x)←0; 7: **for** *b*=1 **to** *V* **do** 8: Draw a bootstrap sample *UN*_*b*_ of size *R* in *UN*; 9: Train a classifier *f*_*b*_ to discriminate *PO* against *UN*_*b*_ with *opPara* and *W*_*op*_; 10: For any $x\epsilon \left(UN\backslash UN_{b}\right)$ , update: 11: $f\left(x\right)\leftarrow f\left(x\right)+f_{b}\left(x\right);$ 12: n(x)←n(x)+1; 13: **end for** **Output** The score $s\left(x\right)=\frac{f\left(x\right)}{n\left(x\right)},for\;x\epsilon \;UN.$

The basic combination of the sub-vectors is to merge the disease gene distribution entropy sub-vector $<V_{d}^{chr}>$ and lncRNA sequence's *k*-mer frequency sub-vector $<V_{Lnc}^{seq}>$ Here, we set *k*=3 (*k*-mer size for lncRNA sequence) and *k*1=9 (*k*-mer size for disease gene series) in the previous section. This basic feature vector is a 109-dimensional (45+64) feature vector, simply denoted by sf1+sf3. We name it the type-0 feature vector. Adding other sub-vectors such as the disease pathway distribution entropy vector $<V_{d}^{path}>$ (sf2, 30-dimensional), lncRNA expression profile $<V_{Lnc}^{prof}>$ (sf4, 16-dimensional), the basic feature vector can be expanded into another three feature types, i.e., the feature type 1∼3 in Table 1. Furthermore, the co-expression feature namely the fifth sub-vector $<V_{co-\exp}>$ (sf5, 3-dimensional) was added to each of the former combinations to form four more feature types which are showed in the last four lines of Table 1.

**Table 1:** Feature types and their corresponding performance

Type	Combination of sub-vectors	Liner kernel	RBF kernel
**0**	sf1, sf3	*C* = 7, F1 = 0.6668	*C* = -1, *G* = -1, F1 = 0.6734
**1**	sf1, sf2, sf3	*C* = 2, F1 = 0.6895	*C* = 3, G = -5, F1 = 0.7024
**2**	sf1, sf3, sf4	*C* = 7, F1 = 0.6692	*C* = 6, *G* = -2, F1 = 0.6734
**3**	sf1, sf2, sf3, sf4	*C* = 0, F1 = 0.6942	*C* = 5, *G* = -7, F1 = 0.7058
**4**	sf1, sf3, sf5	*C* = 8, F1 = 0.6658	*C* = 0, *G* = -2, F1 = 0.6768
**5**	sf1, sf2, sf3, sf5	*C* = 0, F1 = 0.6906	*C* = 4, *G* = -6, F1 =0.7032
**6**	sf1, sf3, sf4, sf5	*C* = 1, F1 = 0.6708	*C* = 0, *G* = -2, F1 = 0.6748
**7**	sf1, sf2, sf3, sf4, sf5	*C* = 2, F1 = 0.7004	*C* = 3, *G* = -5, F1 = 0.7114

Under these different types of vector representation, we tested the liner kernel and RBF kernel with parameter *c* = 2^*C*^, where *C* ranges from -8 to 8 with the step size of 1 (liner kernel and RBF kernel) and *g* = 2^*G*^, where *G* ranges from -8 to 8 with the step size of 1 (RBF kernel). The best 5-fold cross validation results (F1 scores) on the lncRNADisease dataset are shown in the third and fourth columns of Table 1.

Adding the disease pathway distribution entropy sub-vector $<V_{d}^{path}>$ (i.e., sf2) can improve the performance for predicting disease-lncRNA associations (type1 *vs.* type0, type3 *vs.* type2, type7 *vs.* type6, averagely improved by 0.0257 for liner SVM and 0.0307 for RBF SVM respectively). However, the improvement by adding the lncRNA expression profile is not as high as adding the disease pathway distribution entropy sub-vector (0.0052 for liner SVM, 0.0021 for RBF SVM averagely). The co-expression feature vector $<V_{co-exp}>$ can further improve the prediction performance averagely by 0.0039 and 0.0033 for liner SVM and RBF svm respectively. The combination of all the 5 sub-vectors (i.e., the type 7 feature vector) worked the best among the 8 types of feature vectors (on average improving by 0.0223 for liner SVM and 0.0243 for RBF SVM). Furthermore, the RBF kernel outperforms the liner kernel (on average improving by 0.0092). Thus, our baseline classifier is the RBF SVM (*C* = 3, *G* = -5) with the type 7 feature vector representation (*W*=7).

**Figure 5:**
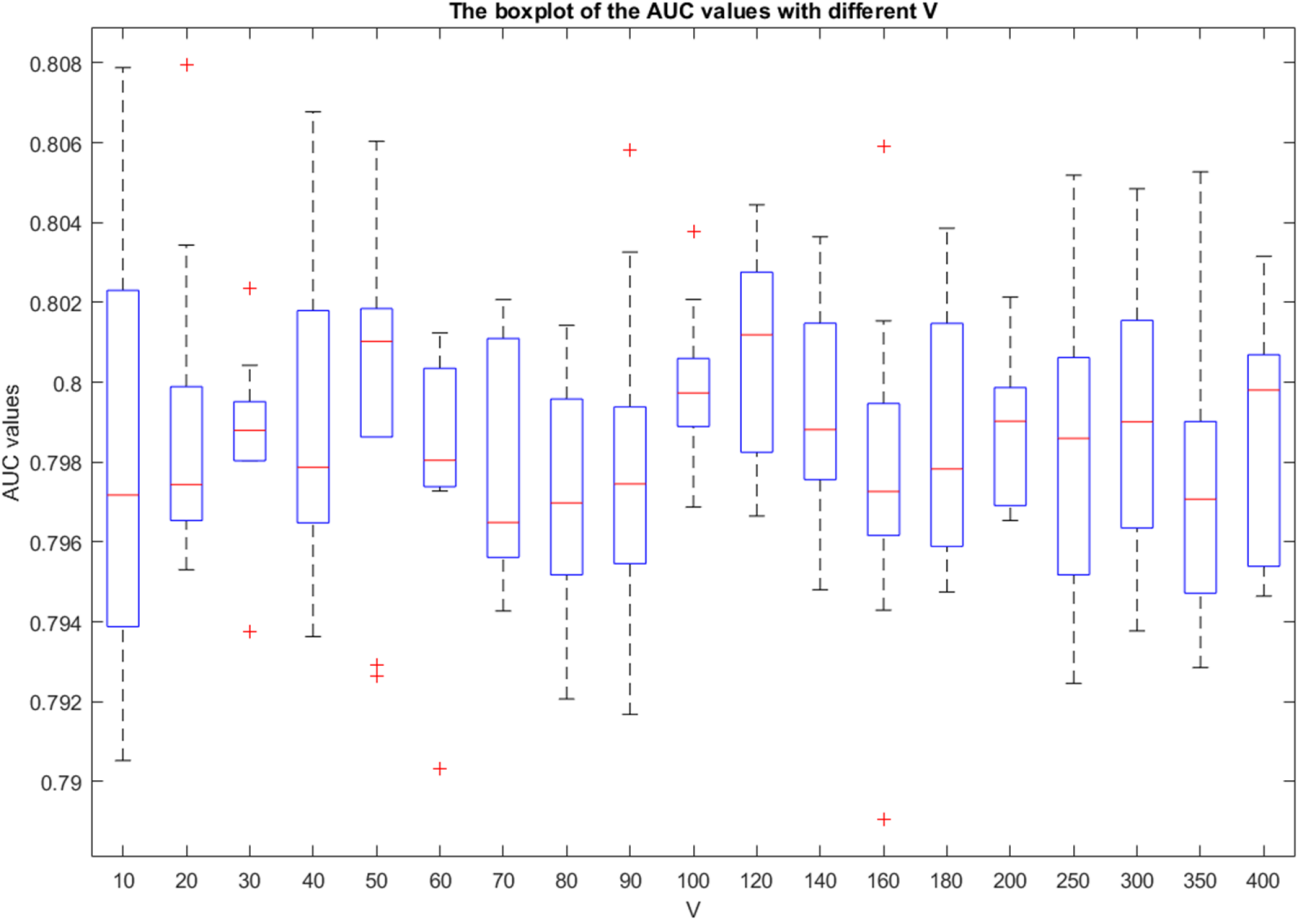
The boxplot graph of the AUC values for the 5-fold cross validation experiments The x-axis is the value of *V*, and the y-axis is the corresponding AUC values. The changes of the AUC values with different *V* are tiny. For a given *V*, the prediction results are stable.

Using all the sub-vectors (i.e., the type 7 feature vector) to represent a pair of disease and lncRNA gene, the 5-fold cross validation AUC results on the lncRNADisease dataset by bagging SVM are shown in Figure 5, using different bootstrap sample size *R* and the bootstrap number *V*. Here, we repeated the experiment 10 times. The AUC values were computed via comparing the scores of known pairs (set to be unknown during the cross validation) with those unknown ones. We note that we simply set *R*=|*PO*| as Mordelet [24] had proved that setting *R* to be the same as the size of positive samples is a safe choice for the bagging SVM.

The AUC values change in a narrow scope (0.79-0.81) when the bootstrap number *V* varies from 10 to 400. In fact, the running time for computing the scores of unknown samples increases significantly when *V* is increasing. As bigger *V* achieves weak improvement of the performance but results in significant increase of time cost, we suggest to fix *V*=10. This is consistent with the conclusion of Mordelet's report that when *R* is large, the SVM usually rarely benefits from bagging. Thus, our final PU learning classifier is built with following parameters: RBF kernel SVM with *C* = 3, *G* = -5, *V* = 10, *R*= |*PO*| and *W* = 7.

### Performance comparison and case studies

**Figure 6:**
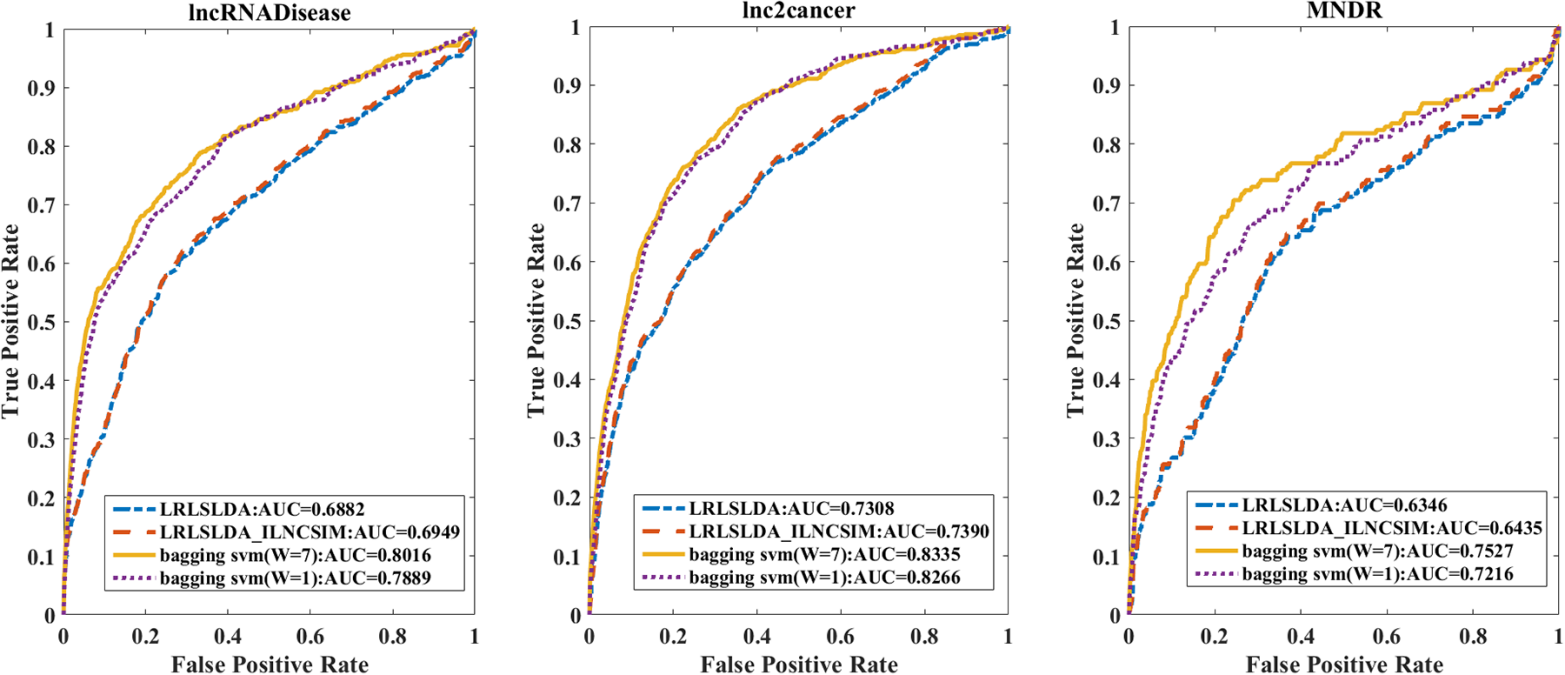
The leave-one-out cross validation results based on three datasets with different methods Four methods were compared, our method with type 7 (*W*=7) feature and type 1 (*W*=1) feature, LRLSLDA method and the LRLSLDA_ILNCSIM method. Our type 7 method works best for all three datasets.

In comparison with two state-of-the-art disease-lncRNA association prediction methods LRLSLDA [14] and LRLSLDA_ILNCSIM [27], our leave-one-out cross-validation AUC performance is much better on the three datasets (Figure 6). We note that the source codes of these two existing methods are not available, but we implemented their algorithms for a fair comparison. Their datasets are not available either.

Our method with type 7 feature vector has a superior performance (AUC=0.8016, 0.8335 and 0.7527 on the three datasets) over the other three methods: the type 1 vector method (AUC=0.7889, 0.8266 and 0.7216), the LRLSLDA (AUC=0.6882, 0.7308 and 0.6346) and the LRLSLDA_ILNCSIM (AUC=0.6949, 0.7390 and 0.6435). We note that our type 1 vector needs just the accessible information such as disease genes and lncRNA sequences, but it can achieve close performance as the type 7 vector method did.

We also did the leave-one-disease-out cross validation when assuming that all the related lncRNAs of a given disease are unknown. Then we computed the possibilities of the lncRNAs to be associated with the disease. The AUC value was used to test how are those already known related lncRNAs ranked comparing with the unknown ones. There are more than 40% (68 out of 162 diseases) of the diseases can achieve an AUC value higher than 0.9. The average AUC of all the diseases is 0.7356. This suggests that our method is capable of predicting disease-lncRNA associations even without knowing any association with a given disease.

**Figure 7:**
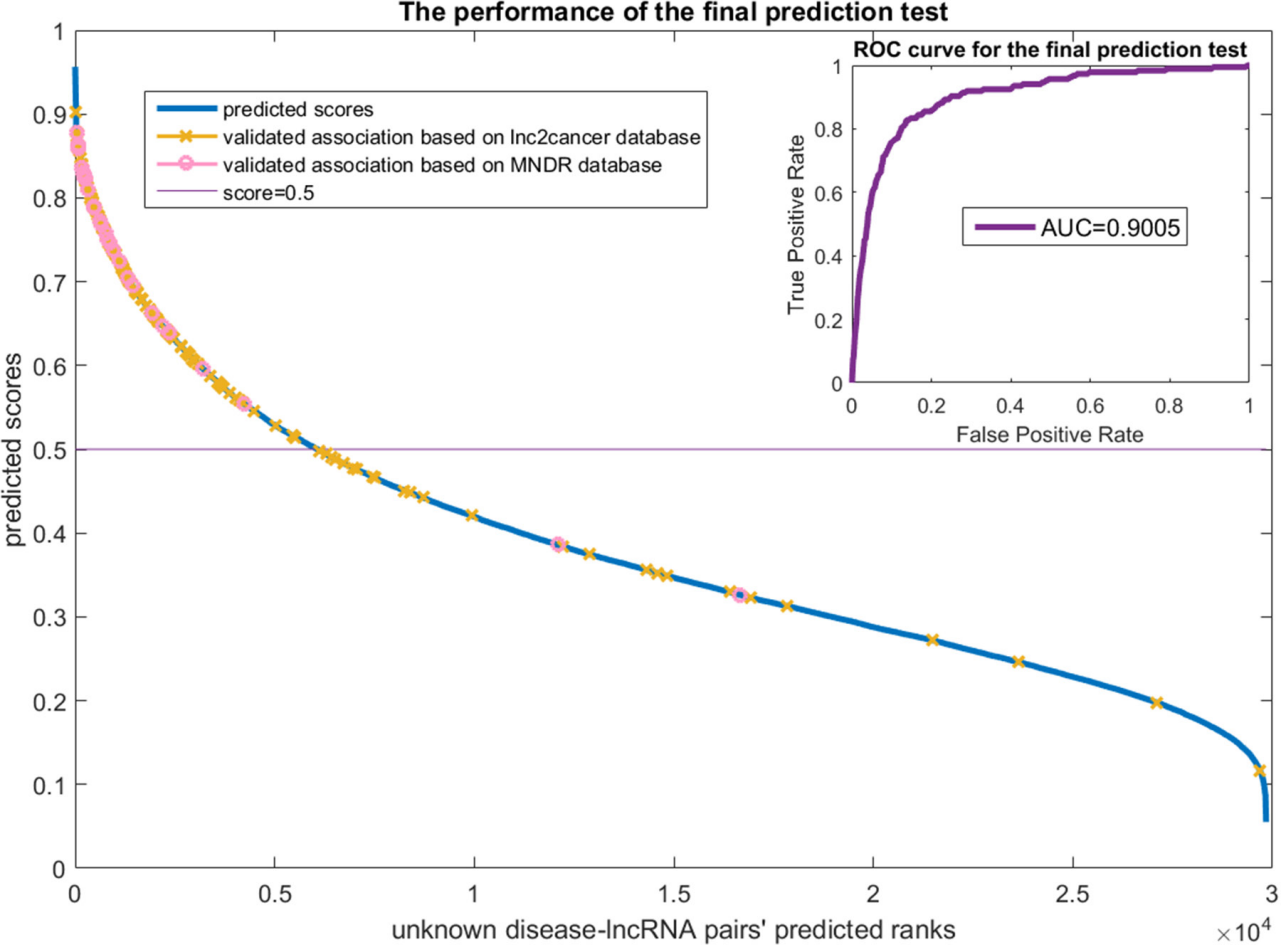
The final prediction test on the lncRNADisease dataset The x-axis is the unknown disease-lncRNA pairs' predicted ranks. The y-axis are the predicted scores which means the possibilities of the samples to be positive. The predicted results were validated via the lnc2cancer and MNDR datasets. The validated samples were marked on the score curve. The ROC curve that compares the scores of the validated samples and the remain unknown samples is drawn at the top right of this figure. The AUC value achieves 0.9005.

We did an experiment to predict disease related lncRNAs using the known 454 positive samples and the 29840 unlabeled samples by PU learning. The predicted results were validated using two other datasets (166 lnc2cancer samples and 29 MNDR samples overlap with the 29840 unlabeled samples). The ranking scores of the 29840 unlabeled samples and a ROC curve are plotted in Figure 7.

Figure 7 shows that most of the validated samples are ranked at good positions. We regarded those 166 lnc2cancer samples and 29 MNDR samples as positive while remaining unknown samples as negative and draw the ROC curve at the top right of Figure 7. It achieves the AUC value of 0.9005, which reveals that our prediction can always rank the positive samples well. We also did case studies for breast cancer and prostate cancer. Breast cancer is the leading type of cancer in women, accounting for 25% of all women cancer patients [28]. Prostate cancer is the second most common type of cancer and the fifth leading cause of cancer-related death in men [28]. We list in Table 2 top 5 lncRNAs that are (possibly) related to these two cancer types.

**Table 2:** Case studies for predicting breast cancer and prostate cancer related lncRNAs

Cancer type	Predicted related lncRNA	Predicted scores	Validated status
breast cancer	UCA1	0.8685	validated by lnc2cancer
breast cancer	DLEU2	0.8375	validated by literatures
breast cancer	EPB41L4A-AS1	0.8356	not validated
breast cancer	LINC00271	0.8297	validated by literatures
breast cancer	7SK	0.8280	validated by literatures
prostate cancer	UCA1	0.9220	validated by lnc2cancer
prostate cancer	BCYRN1	0.8983	not validated
prostate cancer	HOTAIR	0.8952	validated by lnc2cancer
prostate cancer	ZFAS1	0.8810	not validated
prostate cancer	BOK-AS1	0.8800	not validated

The most-top ranked lncNA that is related to breast cancer is UCA1. This annotation has been already recorded in the lnc2cancer database. The second highest ranked lncRNA is DLEU2. In fact, DLEU2 is frequently deleted in malignancy [29]. It functions as a critical host gene of the cell cycle inhibitory microRNAs miR-15a and miR-16-1. Both of these two microRNAs are related to the breast cancer [30]. The 4^th^ and 5^th^ top-ranked lncRNAs LINC0271 [31] and 7SK [32] are related to breast cancers. As to prostate cancer, two top-ranked lncRNAs UCA1 and HOTAIR have been actually stored in the lnc2cancer database. These case studies support that our disease vector representation and PU learning methods are effective to prioritize disease related lncRNA genes.

## MATERIALS AND METHODS

Datasets of diseases and disease related genes were collected and relevant human KEGG pathways were collected as well for the construction of the disease vectorization model and the disease gene prediction method. The details of the datasets and prediction algorithms are presented below.

### Diseases, disease genes and KEGG pathways

The Medical Subject Headings (MeSH) [33], Comparative Toxicogenomics Database (CTD) [34], Disease Ontology (DO) [35] and Online Mendelian Inheritance in Man (OMIM) [36] are widely visited databases containing massive amount of disease related information. However, there is no standard for the adoption of disease names or ids between these databases. We mapped disease names to DO ids using the DO, MeSH and CTD as dictionaries. Similarly, for genes, we did id or name conversion using the data records from the HUGO Gene Nomenclature Committee (HGNC) [37] database. It contains reference records of genes among a great number of widely used databases. In this work, we mainly mapped the genes obtained from various resources to entrez gene ids [38]. We downloaded the HGNC database on Jun 17, 2016. There are totally 39670 approved gene records with entrez gene ids including 19025 protein coding genes and 20645 non-protein coding genes.

We downloaded disease-gene associations from the supplementary file of a published article [18] which contains 117,190 associations between 2817 diseases and 12063 genes. The authors collected these data records from database SIDD [39]. Each of the diseases has a unique id from database DO. After data correction and redundancy removal according to the latest version of the databases DO, MeSH, CTD and HGNC, we obtained a set of 114754 disease-gene associations between 2802 diseases and 10893 genes (including 10321 protein coding genes and 572 non-protein coding genes). The human KEGG pathways were extracted from the KEGG database on June 21, 2016. There are 303 unique pathways containing 7060 unique genes (all have an entrez gene id). All these datasets are listed in Supplementary File 3 and Supplementary File 4.

### Associations between diseases and lncRNAs

The disease-lncRNA associations were obtained from three databases: lncRNADisease (downloaded on April 18, 2016), lnc2cancer (downloaded on July 4, 2016) and MNDR (downloaded on June 30, 2016). There are 1102, 1239 and 754 disease-lncRNA associations (redundant and unclear information are existing). For the diseases, we mapped them to DO. To construct our PU learning model for disease related lncRNA prediction, we collected the sequences and expression profiles of the lncRNAs.

We mapped each of these lncRNAs to its corresponding ensembl gene id, RefSeq accession id, entrez gene id and other detail information. This process was manually finished via searching and comparing the lncRNA related databases such as ensembl [40], NONCODE [41], Lncipedia [42], lncRNAdb [43], and HGNC. Then, lncRNA sequences were extracted from the RefSeq [44]. We downloaded the expression level of 60245 genes (coding or non-coding genes matched with an ensembl id and gene symbol) in 16 tissues from the Expression Atlas [45].

Finally, we obtained 454 disease-lncRNA associations from lncRNADisease (between 162 diseases having known disease genes and 187 lncRNAs with known sequences and expression levels). Those 594 (79 cancers, 310 lncRNAs) and 176 (86 diseases, 57 lncRNAs) more pairs were extracted from lnc2cancer and MNDR respectively. For those diseases that not exist in the above 2802 ones, disease genes were obtained from the CTD, DisgeNet [46], OMIM and malaCard [47]. The datasets are stored in Supplementary File 5.

### Disease gene chromosome preference analysis and disease vectorization method

Human genes are located on mitochondrion and 24 unique chromosomes including 22 autosomes and two sex chromosomes. The genes’ locations on the chromosomes or mitochondrion have been labeled by the HGNC database. As disease related genes are distributed at varies locations and have a different number of each disease, we hypothesize that the gene distribution differences between diseases on the chromosomes or mitochondrion may reflect the divergences of the diseases. We also hypothesize that disease genes may have some preferred chromosomes for some diseases. This hypothesis can be investigated by the disease genes’ chromosomes enrichment analysis via fisher's exact test [48]. Thus, it is better to characterize the distribution properties of disease genes on each of the chromosomes instead of on the whole known gene set (we call it a “part overcomes the whole” hypothesis).

On the basis of these hypotheses, we considered to vectorize a disease via modeling the distribution property of its related gene set. However, with just the gene distribution characteristics, there may be no gene function information involved. Thus, we considered to extract the distribution properties of disease gene enriched KEGG pathways comparing to all the known pathways to inject complementary information for our vector representation of diseases. This vectorization process includes the following steps:

Step1: Initialization. Sorting all known genes according to their chromosome locations and sorting all the human KEGG pathways by their ids.

Step2: Grouping. Dividing the genes and pathways into groups. Producing a status series for each group with the length equals to the number of genes or pathways it contains. These statuses are initialized to be 0 (inactivated).

Step3**:** Mapping. For a given disease related gene set, mapping it to the gene groups and mapping its enriched pathways to the pathway groups. Then, setting the corresponding status of a gene or pathway in the status series to be 1 (activated) if it has been mapped.

Step4**:** Vectorization. Calculating the status series’ *k*-mer information entropy of each gene group or pathway group to quantify them and constructing two sub-vectors for a given disease.

Here, dividing all the genes and pathways into groups is to apply our “part overcomes the whole” hypothesis. In our Results section, we demonstrate that this strategy (part) is more effective for characterizing diseases comparing to the status series without dividing (the whole). As a chromosome always contains a p-arm and a q-arm, we divide the genes into groups according to the natural chromosome substructures. For the pathway status series, we divide it into T groups on average. (There is no guidance for us to divide pathways similar to chromosome structure). Finally, this vectorization model includes two parts: disease gene set vectorization and disease gene enriched pathway set vectorization.

Let *d* represent a disease, and *d*_*g*_ = {*g*_1_, *g*_2_, …, *g*_*c*_, …, *g*_*n*_} be its related gene set. Let all of the approved genes from HGNC be *G* = {*g*_1_, *g*_2_, …, *g*_*i*_, …, *g*_*N*_}, and the pathway set from KEGG database be *P* = {*p*_1_, *p*_2_, …, *p*_*j*_, …, *p*_*M*_}. Let the unique genes in *P* be represented as *G*_*p*_ = {*g*_1_, *g*_2_, …, *g*_*A*_} while each pathway related gene set as *g*_*pj*_. We define a *k*-mer sub-status series as (*s*1, *s*2, …, *s*_*r*_, …, *s*_*k*_), where *s*_*r*_ = 0 or 1. By definition, there can be 2^*k*^ possible *k*-mer sub-status series. The detail process is described as a pseudo codes in Algorithm 1 and outlined in Figure 1. The source codes can be referred to Supplementary Codes.

The outputs of our vectorization algorithm are two fix length vectors: the disease gene distribution entropy vector $<V_{d}^{chr}>$ with length of *S* and the disease pathway distribution entropy vector $<V_{d}^{path}>$ with length of *T*. To test whether our disease vectors are effective and to determine the parameters such as the *k*-mer sizes, we apply them to compute the disease similarities. For two given diseases *d*1 and *d*2, we derive their disease gene distribution entropy vectors $<V_{d1}^{chr}>$, $<V_{d2}^{chr}>$ and their disease pathway distribution entropy vectors $<V_{d1}^{path}>$, $<V_{d2}^{path}>$ with Algorithm 1. Then we compute the similarity between *d*1 and *d*2 with their vectors. The similarity between *d*1 and *d*2 is denoted as *Sim* (*d*1, *d*2) and computed by:

$$\left\{ \begin{array}{c} simGe\left(d1,d2\right)=0\;if\|V_{d1}^{chr}\|\times \|V_{d2}^{chr}\|=0;\\ subspace\left(V_{d1}^{chr},V_{d1}^{chr}\right)\;else \end{array}\right.$$ (1)

$$\left\{ \begin{array}{c} simPe\left(d1,d2\right)=0\;if\|V_{d1}^{path}\|\times \|V_{d1}^{path}\|=0;\\ subspace\left(V_{d1}^{path},V_{d1}^{path}\right)\;else \end{array}\right.$$ (2)

$$Sim\left(d1,d2\right)=e^{-\left[\theta \times simGe+\left(1-\theta \right)\times simPe\right]}$$ (3)

where *θ* is a parameter to mediate the ratio of each vector's contribution to the similarity. || ● || means the norm. *subspace*(*x*, *y*) is the function to obtain the angle between two vectors *x* and *y*. A larger value of *Sim*(*d*1, *d*2) shows more similar between the two diseases.

The four parameters *k*1(the size of *k*-mer for gene series), *k*2 (the size of *k*-mer for pathway series), *T* and *θ* can be determined via a performance test through comparing the disease similarity with a benchmark dataset. We first set *θ*=1 to optimize *k*1 and set *θ*=0 to optimize *k*2, *T* with the objective of achieving the best performance. Then, the three parameters are set as the optimal values to select the best *θ*. Similarly, we can also apply subspaces between the disease gene status series (a disease is represented as a fixed-length vector with the elements equal to 0 or 1) or the pathway status series themselves instead to measure the similarities of diseases. We call them the disease gene status series vector method and the pathway status series vector method. In the Results section, we compare the performances of our status series methods and our entropy vector methods to prove our “part overcomes the whole” hypothesis.

### Prioritizing disease related lncRNA genes

We always just have the positive samples of disease-lncRNA associations, as the negative samples, namely the lncRNAs that do not relate to the diseases, are neglected or even cannot be obtained. Supervised learning algorithms are unable to deal with these situations. However, the Positive Unlabeled learning (PU learning) method [49] can address this issue effectively. PU learning has been an effective method for solving similar problems in bioinformatics such as disease gene prediction [50], predicting conformational B-cell epitopes [51], splicing elucidation [52] and kinase substrate prediction [53]. These PU learning approaches are mainly derived from two types of PU learning algorithms: the biased-svm [54] and Elkan *et al*'s lemmas [55]. The application of Elkan *et al*'s lemmas requires the satisfaction of “selected completely at random assumption”, while the biased-svm methods need to tune a set of parameters. Mordelet *et al* [24] proposed a bagging svm model for PU learning and proved that their model can match and even outperform the biased-svm algorithm. Especially, the bagging svm for PU learning algorithm can run considerably faster. We adopt this bagging svm PU learning to prioritize disease related lncRNA genes.

Let *Lnc* be a lncRNA gene, represented as *Lnc*=*l*_1_*l*_2_… *l*_*e*_ *…l*_*O*_. We calculate its *k*-mer frequency $<V_{Lnc}^{seq}>$ and its expression profile $<V_{Lnc}^{prof}>$. As there are four kinds of nucleotides in a lncRNA sequence (i.e., *l*_*e*_ ϵ {A, G, C, T} ), there are 4^*k*^ possible *k*-mers. These *k*-mers are sorted by their alphabetic order. Their frequencies are counted via the window sliding mechanism with the window size of *k* and a step size 1, which are then the elements of the vector $<V_{Lnc}^{seq}>$. The expression profile of *Lnc* can be extracted from the Expression Atlas [45]. The expression levels of the lncRNA gene in the 16 tissues are the elements of the vector $<V_{Lnc}^{prof}>$.

Then for a disease-lncRNA pair, *e.g. d*-*Lnc*, we construct another feature namely vector $<V_{co-\exp}>$, called the co-expression levels. This sub-vector can be constructed on the basis of the principle that a disease related lncRNA gene may show the preference of co-expressing with other genes associating with this disease (such as the lncRNA’s targets). This sub-vector contains three elements, *i.e.* the maximum, minimum and average spearman correlation coefficients $\left(<V_{co-\exp}>=<{max}_{co-exp},{min}_{co-exp},avg_{co-exp}>\right)$ between the expression profile of *Lnc* and all the known disease *d* related genes’ expression profiles.

The whole disease-lncRNA feature vector is formed by combining the five sub-vectors: the disease gene distribution entropy vector $<V_{d}^{chr}>$ (sf1), disease pathway distribution entropy vector $<V_{d}^{path}>$ (sf2), lncRNA sequence's *k*-mer frequency $<V_{Lnc}^{seq}>$ (sf3), lncRNA expression profile $<V_{Lnc}^{prof}>$ (sf4), and the co-expression features $<V_{co-exp}>$ (sf5). The pseudo codes for prioritizing the disease related lncRNAs with the bagging SVM for PU learning model are shown as Algorithm 2.

In Algorithm 2, |*PO*| means the sample size of the positive dataset. The feature type means the type of combination of the five sub-vectors. The feature vector $<V_{d}^{chr},V_{Lnc}^{seq}>$ is used as the basic feature type. Adding the remaining sub-features to this basic type makes new feature types. The best one can be identified via comparing the results of the cross-validation experiments. After obtaining the scores for the unlabeled samples, we sort them. The larger scores imply that the samples are more likely to be positive ones.

## CONCLUSION

In this article, we proposed a novel disease vectorization representation and applied for a PU learning method to predict and prioritize disease related lncRNA genes. A disease is newly characterized here using the distribution properties of disease genes on the chromosome substructures and its related KEGG pathways to all the pathways. Our vectorization model can be applied to compute the disease similarities effectively. Testing on the benchmark datasets, our method can work better than the state-of-the-art methods. Especially, it can also work with only lncRNA sequences information or without known related association.

Future work has been planned to improve the performance of our vectorization model. First, more accurate disease genes will be collected as our model critically relies on the reliability of disease genes. Secondly, more information will be introduced to decrease the disease gene dependency such as the disease symptom, the disease semantics and so on. Furthermore, the relationship between the disease genes and lncRNA targets will be considered to extract more effective features to predict disease-lncRNA gene associations.

## SUPPLEMENTARY MATERIALS FIGURES AND TABLES












