# Supplementary material for: Chromosome preference of disease genes and vectorization for the prediction of non-coding disease genes
Source: Oncotarget. 2017 Aug 24;8(45):78901–16. doi: 10.18632/oncotarget.20481 (PMC5668007; doi:10.18632/oncotarget.20481)
Supplement: Supplementary file 1 [file oncotarget-08-78901-s001.pdf]

Chromosome preference of disease genes and vectorization for the prediction of non-coding disease genes

SUPPLEMENTARY MATERIALS

Introduction of supplementary files

There are four supplementary files and a file folder for supplementary codes. Supplementary file 1 gives a simple description of the supplementary files and illustrates some complementary results such as the disease gene enriched pathway statistic results and the comparison of our method and Cheng *et al*. [1] method for computing disease similarities on the original benchmark dataset. In the Supplementary File 2, we provide the original 2802 disease list and the 70 original benchmark similar disease pairs. Zhou's [2] HSDN mapped diseases together with the 56 mapped similar disease pairs are also provided. Supplementary File 3 and Supplementary File 4 list the disease genes, human pathways, pathway associated genes and all the protein coding genes for our disease vectorization. The Supplementary File 5 shows three disease-lncRNA association datasets and the gene expression profiles of 60245 genes are shown. Supplementary Codes are the MATLAB codes for implementing our disease vectorization method and our PU learning for predicting disease-lncRNA gene associations.

SUPPLEMENTARY FILE 1

Pathway enrichment analysis for understanding the distribution of disease genes in human pathways

we considered to extract the distribution properties of disease gene enriched KEGG pathways comparing to all the known pathways to inject complementary information for our vector representation of diseases. We first did the disease gene enrichment analysis with the fisher exact test with the threshold of p-value<=0.05 on 2802 diseases. Then, we did the statistics about the distribution of disease genes in totally 303 human pathways. The pie chart in Supplementary Figure 1 shows our statistics about how many pathways can be enriched by the diseases' genes. According to the number of those 2802 diseases' genes enriched pathways, we classify them into five classes such as extreme complex diseases which associate with no less than 100 pathways, those relative complex diseases are the ones with their genes enriched to no less than 50 pathways but less than 100 pathways. Diseases with their genes can be enriched to the number of pathways in the bound of [10,50) are medium complex diseases. Those just relate to more than 2 but less than 10 pathways are relative simple diseases while other diseases just relate to no more than 1 pathway are simple disease or unknown type diseases. From the pie chart, we can see that medium complex diseases and relative simple diseases are the two biggest disease classes with about 28% and 37% of all the 2802 diseases. Meanwhile, those extreme complex diseases and simple diseases and unknown type diseases just contain 4% and 14% of those 2802 diseases respectively. Complex diseases that associated with more than 10 pathways (about 57%) are more common that those simple diseases that relate to no than 10 pathways (about 42%).

In addition, we also analyzed how the diseases are distributed on each of the pathways. In Supplementary Figure 2, we show the frequencies of the diseases that enriched to the pathways (we call those disease gene enriched pathways as those diseases enriched pathways for convenience). We also can find that each pathway is enriched by various number of diseases. In other words, the pathways are unevenly enriched by the diseases.

In the top right of the Supplementary Figure 2, we also classify the pathways into five classes such as the Phenotype Extreme Highly Enriched pathways (PEEP) where more than 30% of the 2802 diseases' genes can be enriched to it; Phenotype Highly Enriched pathways (PHEP) where no less than 20% but less than 30% diseases' genes are enriched to it; Phenotype Relative Highly Enriched pathways (PREP) with the percent of diseases belongs to [10%, 20%) can be enriched to it; Phenotype Relative Low Enriched pathways (PRLEP), it relates to [1%, 10%) of diseases; and Phenotype Low Enriched pathways (PLEP) when no more than 1% of the diseases associate with it. We can see that 139 pathways are PRLEP which accounts for nearly a half of all the pathways (139 vs. 303). Just 6 pathways can be enriched by more than 30% of the diseases (PEEP). These pathways are more likely to be interrupted and may be also regarded as the target pathways for disease treatment. Both PREP and PLEP contains 61 pathways while the PHEP has 36 pathways. This means that the diseases may also have pathways preferences as to the chromosome preferences. This property also give us the inspiration of characterizing diseases with their pathways enrichment features. The pathway also reflects the function of genes and we can use

this vector to introduce complementary information for the divergence of diseases.

Comparison of different methods for computing disease similarities

We tested the performance of our vectorization model for computing disease similarities on the downloaded dataset from the supplementary files of Cheng's paper. It contains a candidate disease set and a benchmark set of similar disease pairs. The disease set is composed of 2802 diseases and their related genes. There are 70 similar disease pairs in the benchmark set. Following cheng's method, we draw a ROC curve to display how our method can rank these similar pairs comparing with those randomly selected unknown disease pairs. That means, for a given threshold, if the similarity of a pair in the benchmark set exceeds the threshold, it will be a true positive, otherwise, a false negative. Inversely, an unknown disease pair exceeds the threshold will be a false positive. During the process, 700 testing disease-disease pairs will be randomly selected from the candidate diseases (no overlapping between testing pairs and benchmark set). This process was repeated 100 times. The ROC curves are shown in Supplementary Figure 3.

The comparison results show that the integrated feature based method overcomes the other methods including our pathway entropy vector method and the disease gene set entropy vector method. However, it just improves 0.0029 comparing with just disease gene set entropy based method (k1=9, θ=1). It implies that there are not much complementary between the disease gene set entropy and gene enriched pathway entropy features as to compute the similarities of diseases. Our entropy vector methods are all overcomes the pathway status series and the gene status series method. Thus, our diving the genes and pathways into groups and computing the entropies of them is an effective strategy for representing diseases. Our method also outperforms cheng's method as to predict similar disease pairs.

Comparison of different methods for predicting disease-lncRNA associations

In our main manuscript, we compared our bagging SVM methods for predicting disease-lncRNA relationships with other two existing methods such as the LRLSLDA [3] and LRLSLDA-ILNCSIM [4] on three datasets. We also compared our bagging svm method with the bagging KNN method. Here, the bagging KNN is constructed similar to bagging svm where the classifier svm is replaced with the KNN [5]. We kept the other parameters the same with bagging svm and tuned the parameter K for KNN (the number of nearest neighbors) on the lncRNADisease dataset. We set K=3 to 15 with step 2. The type 1 (w=1) and type 7 (w=7) features were tested. The following bar graph shows the overall AUC values with different K.

When K>7, the performance changes little for both w=1 and w=7. Thus, we set K=11 as the best parameter. The ROC curves of the bagging KNN method, our original bagging svm method and the other two existing methods were compared. The following Supplementary Figure 5 describes the ROC curves for different methods testing on the three datasets.

Both the bagging KNN and the bagging svm methods work better than those existing non-vector computation methods for prioritizing disease-lncRNA associations. The bagging svm works best among all of them. On the lncRNADisease dataset, the bagging svm achieved the AUC value of 0.8016 with the type 7 features where the bagging KNN obtained 0.7599 with the same feature type. For the lnc2cancer and MNDR dataset, the AUC values of the bagging svm and bagging KNN with the type 7 features are: 0.8335,0.9074 for lnc2cancer and 0.7527, 0.7073 for MNDR. Both of these two methods worked better than the LRLSLAD and LRLSLDA-ILNCSIM. These results also proves the advantage of our bagging svm methods for the prediction of disease-lncRNA associations.

REFERENCES

1. Cheng L, Li J, Ju P, Peng J, Wang Y. SemFunSim: a new method for measuring disease similarity by integrating semantic and gene functional association. PloS one. 2014; 9:e99415.
2. Zhou X, Menche J, Barabási AL, Sharma A. Human symptoms–disease network. Nature communications. 2014; 5.
3. Chen X, Yan GY. Novel human lncRNA–disease association inference based on lncRNA expression profiles. Bioinformatics. 2013: btt426.
4. Huang YA, Chen X, You ZH, Huang DS, Chan KC. ILNCSIM: improved lncRNA functional similarity calculation model. Oncotarget. 2016.
5. Peterson, LE. K-nearest neighbor. Scholarpedia. 2009; 4:1883.

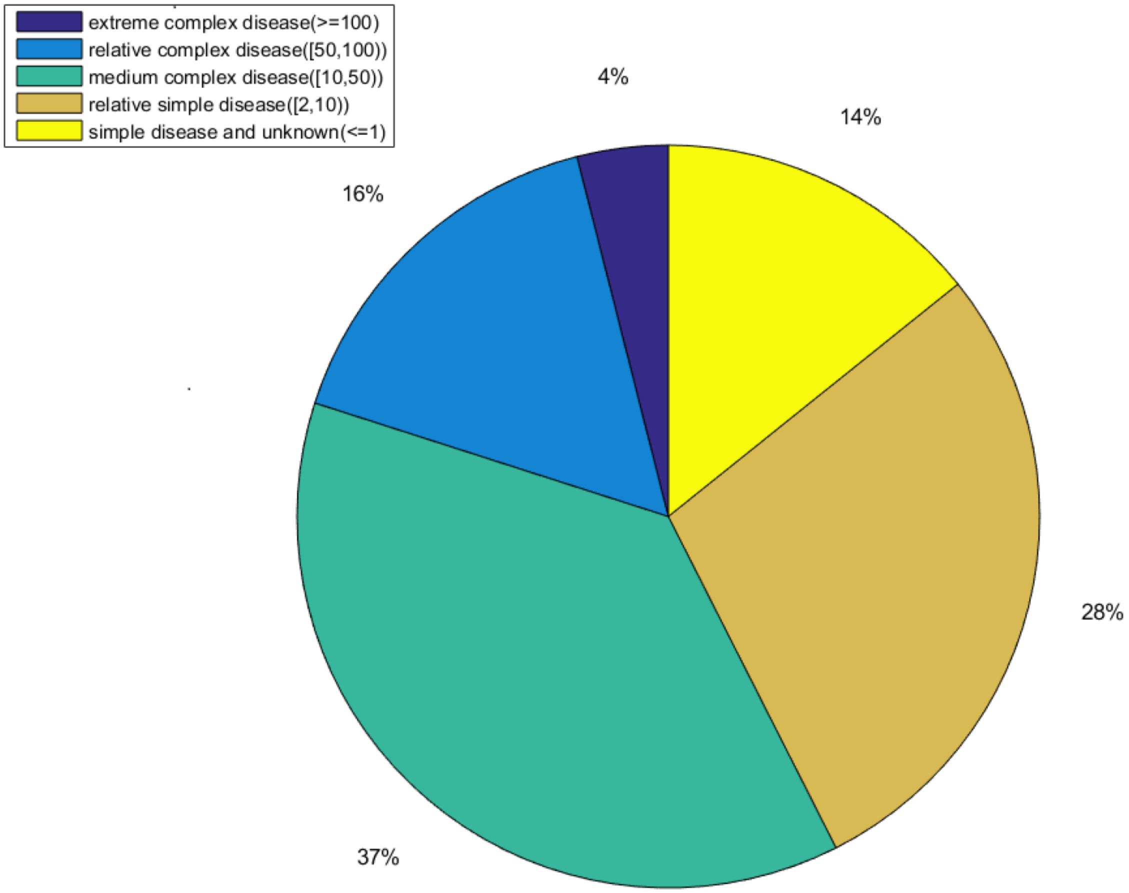

Supplementary Figure 1: The distribution of disease enriched pathways. In this pie chart, we classify the diseases into five classes such extreme complex disease if its genes can be enriched to no less than 100 pathways. Diseases associate with no less than 50 but less than 100 pathways are relative complex diseases. Those diseases with the number of pathways belong to [10,50) are medium complex diseases. Those just relate to more than 2 but less than 10 pathways are relative simple diseases and other diseases just relate to no more than 1 pathway are simple disease or unknown type diseases.

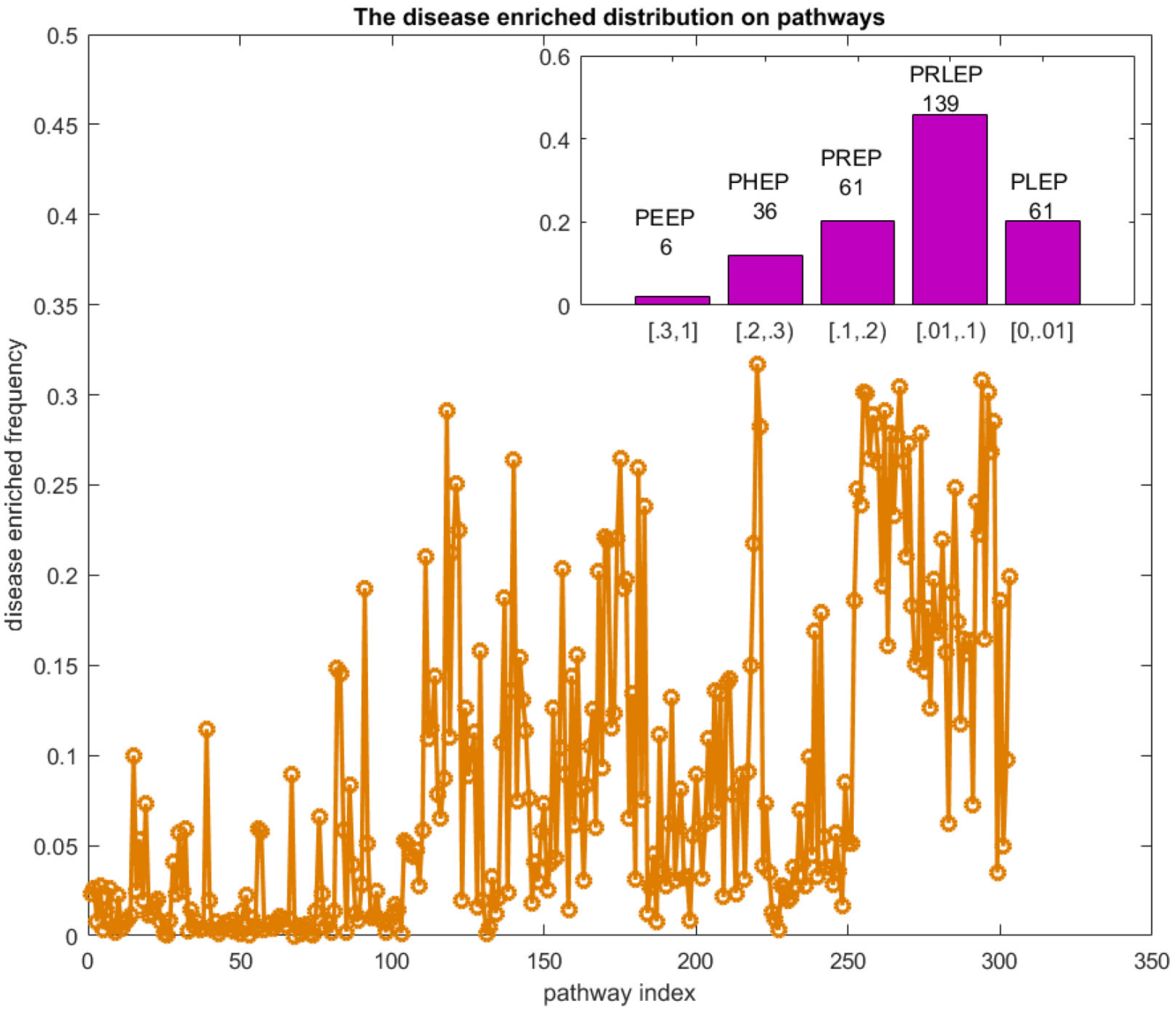

Supplementary Figure 2: The disease enriched frequencies of those pathways and the statistics. The x-axis shows the index of pathways that sorted by their IDs. The y-axis is the frequencies that the disease enriched to each of the pathways. The top right bar graph is to classify pathways into five classes and does the statistics that how many pathways belong to the pathway class. The x-label is the pathway groups and their definitions such as those pathways related to more than 30% of the 2802 diseases are classified as Phenotype Extreme Highly Enriched pathways (PEEP). Pathways associated with 20% to 30% of the diseases are Phenotype Highly Enriched pathways (PHEP), those pathways that enriched by 10%~20% of the diseases are Phenotype Relative Highly Enriched pathways (PREP). Then those pathways enriched by 1%~10% and no more than 1% of the diseases are Phenotype Relative Low Enriched pathways (PRLEP) and Phenotype Low Enriched pathways (PLEP). The y-axis is the percent of the pathways in each class.

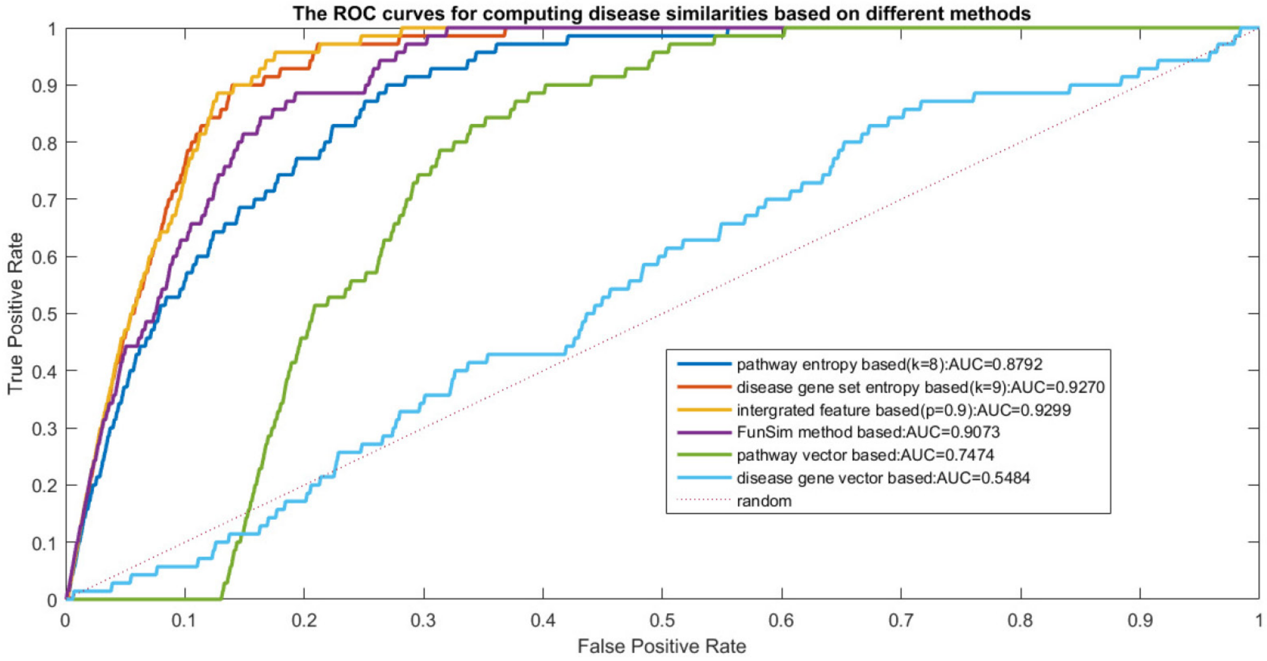

Supplementary Figure 3: The ROC curves of different methods for computing the disease similarities test on 2802 diseases and 70 benchmark similar disease pairs. Here, the ROC curves of totally 6 different methods are showed. The gene enriched pathway entropy based method (θ=0, k=8 AUC=0.8792); the disease gene set entropy based method (θ=1, k=9, AUC=0.9270); the integrated feature method (θ=0.8, AUC=0.9299); the pathway status series based model(AUC=0.7474); the disease gene status series based method (AUC=0.5484) and FunSim (AUC=0.9073).

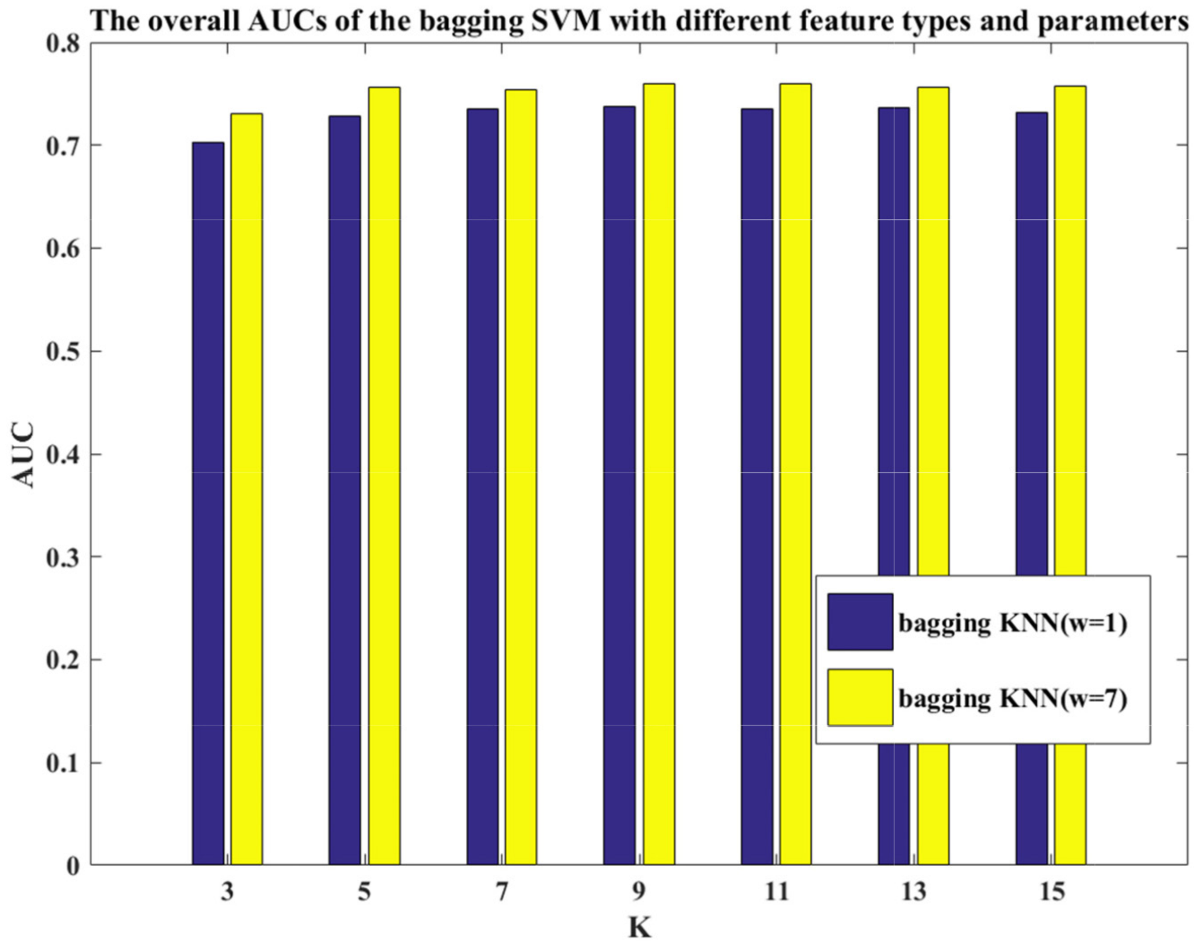

Supplementary Figure 4: The AUC values of the bagging KNN method for predicting disease-lncRNA associations on lncRNADisease dataset. The x-axis is the parameter *K* and the y-axis is the AUC value. We can find that when K>7, the AUC value nearly keeps the same when *K* increases. Thus, we set *K*=11 as the optimal parameter.

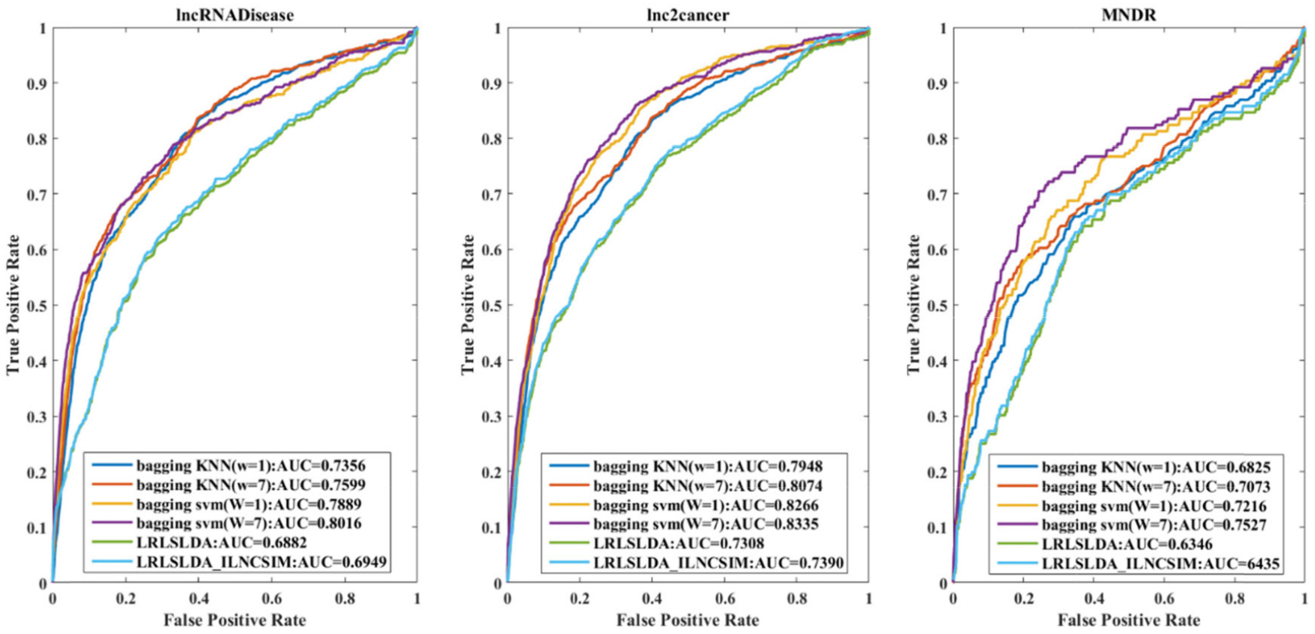

Supplementary Figure 5: The leave-one-out cross validation results based on three datasets with different methods. The KNN methods, SVM methods, LRLSLDA method and the LRLSLDA_ILNCSIM method were compared. Our type 7 method works best for all three datasets. The KNN methods all work better than the other existing methods.

Supplementary File 2: Disease datasets for the disease vectorization analysis. These datasets include the original 2802 diseases, the 70 original benchmark similar disease pairs, Zhou's HSDN mapped diseases and the corresponding 56 mapped similar disease pairs.

See Supplementary File 1

Supplementary File 3: Disease genes for the disease datasets. This table lists all the disease genes corresponding to the diseases in the above disease datasets.

See Supplementary File 2

Supplementary File 4: Human KEGG pathways, pathway genes and protein coding gene information. This table shows the KEGG pathways for human, the pathway related genes and the protein coding genes for the disease vectorization.

See Supplementary File 3

Supplementary File 5: Validated disease-lncRNA association datasets and gene expression profiles. There are three known disease-lncRNA association datasets and a set of gene expression profiles.

See Supplementary File 4

Supplementary Codes: Implemented codes for our disease vectorization and disease-lncRNA association prioritizing algorithms. This file contains all the implemented codes and the related datasets.

See Supplementary File 5
